# The limitations of narrative medicine

**DOI:** 10.1007/s11017-025-09713-6

**Published:** 2025-04-16

**Authors:** Rajeev Dutta

**Affiliations:** School of Medicine and Department of Philosophy, University of California, 85 Humanities Instructional Building, Irvine, CA 92697 USA

**Keywords:** Narrative medicine, Narrative knowledge, Active listening, Empathy, Epistemology

## Abstract

Narrative medicine has emerged over the past few decades as an exciting approach to medical practice, interweaving the practice of medicine with the practices of literary analysis and reflective writing. It is often claimed that narrative medicine enables practitioners to understand and empathize with patient stories, effectively ‘joining’ patients in illness. However, I argue that there are reasons to be suspicious of narrative medicine’s ability to promote patient-centered care. I begin by questioning the distinctiveness of narrative knowledge, suggesting that it is neither able to be propositional knowledge (‘knowledge-that’) nor phenomenal/experiential knowledge (‘knowledge-what-it’s-like’). Then, I consider an alternative reading of narrative medicine, by which narratives are simply ways to structure patient information so that a physician can more readily empathize with the patient. I dismiss this alternative as unsatisfactory given that it depends on either all patients building narratives or physicians imposing narrative structure(s) where one does not inherently exist, thus overriding patients. Finally, I provide possible supplements and alternatives to narrative medicine, proposing that active listening and the removal of systemic barriers to physicians’ abilities to provide humanistic care (e.g., lower administrative, profit, and documentation burdens) may be a first step to putting empathetic patient care on the forefront. Ultimately, I think that these efforts (while their fruition may present difficulty), rather than sifting through patient information to construct and elevate narratives, present the opportunity to accurately refocus patient-centered care.

## Narrative medicine and its scope

Narrative medicine has characterized creative and reflective efforts in medical humanities since the turn of the century. It is difficult to overstate the spread of the concept of narrative medicine in medical education and practice over the past twenty years. The goals of narrative medicine, as described by Rita Charon, include those of physicians developing the narrative competence to understand and empathize with patient stories, pursuing reflective writing to make sense of physician–patient, physician-self, physician-peer, and physician-society relationships, among others [1]. The implementation of narrative medicine in healthcare settings has demonstrated some potential to enhance the conscientious practice of medicine, enabling humanistic analysis of patient stories, potential phenomenological insight into healing experiences, and the careful approach of unfamiliar contexts with humility.

However, it is critical to acknowledge the limitations of narrative practices. Specifically, medical practitioners should recognize whether the pursuit, apprehension, and transmission of patient narratives actually promotes patient-centered care. Relatedly, in this article, I will discuss what I take to be serious limitations of narrative medicine, describing the ways in which it may be detrimental to empathetic identification with patients on account of its unclear aims and reaches. I propose that the assumption that one can learn about a patient or the phenomenological aspects of illness (i.e., ‘knowledge-what-it’s-like’) by reading or hearing a narrative (let alone a physician’s written account) is immediately suspicious, and one should be skeptical of narrative structure(s) as a substitute for deep, active engagement with a patient, however the patient may present themselves. Further, I argue that narrative medicine’s application to genuine understanding of patients is dubious at best and overriding at worst. For example, the imposition of narrative structure (derived from primarily Western traditions of storytelling, Western norms of relevant narrative elements, etc.) onto a diverse range of patients threatens to erase, or at least mute, the real richness and diversity of the therapeutic relationship. In other words, the best way to learn meaningfully about the patient’s experience is to listen to the patient, rather than clinging to narrative structure (or worse, imposing a narrative structure on information provided by a patient).

Of course, there are other relevant critiques of narrative medicine. For example, it has been argued that a framework of narrative medicine puts constraints on patient presentation of illness, burdening the ill with an additional responsibility of presenting a redemptive story [2]. It has also been argued that narrativity is not as central to human experience as narrative medicine tends to assume and that the benefits of narrative framing of illness experiences are overstated [3, 4]. My critique will integrate some of these threads while merely nodding to others, painting a wide picture of narrative medicine’s limitations.

To be clear, my intention is not to disavow narrative medicine in its entirety: Rather, I insist that one recognize the areas in which narrative medicine does indeed impart the “skills of recognizing, absorbing, interpreting, and being moved by the stories of illness” [5, p. 4]—and crucially, from *which perspective* these skills are deployed—and recognize the areas in which narrative medicine cannot, by itself, afford empathetic insight into the plights of others (and may rather be harmful). For these areas, particularly in the understanding the direct experience of illness, I offer alternative strategies for approaching the goals that narrative medicine has aspired, but by its constitution fails, to attain.

In this paper, I will consider what narratives and narrative knowledge are, discussing what Charon has in mind for them and where (I argue) they cannot succeed in her designs (Sect. "What is a narrative? What is narrative knowledge?"). Then, I will consider an alternative reading of narrative medicine by which narratives make attainable certain kinds of empathy, which I argue is ultimately unsatisfying since it focuses on the physician perspective rather than patient-centered care (Sect. "Whose narrative?"). Finally, I will suggest supplements, and then an alternative of active listening, to narrative medicine to promote empathetic patient-centered care, which may also promote the well-being and capacities of physicians (Sects. "Narrative-listening or active-listening?" and "Empathy and active-listening"). This alternative of active listening requires the removal of obstacles physicians face—such as labor conditions—to be effective, ideally through reducing administrative and documentation burdens.

## What is a narrative? What is narrative knowledge?

While there are numerous accounts of what narratives are (many of which Charon cites; [6–8]), I will focus on Charon’s account of narratives and narrative knowledge.

In numerous places in her book, Charon describes the characteristics of narratives (e.g., temporality, singularity, causality/contingency, intersubjectivity, and ethicality). However, at the outset, she writes that narratives “can be *defined* as stories with a teller, a listener, a time course, a plot, and a point” ([5, p. 3], emphasis mine). This definition seems suitable: it is not too minimal (e.g., as defining narrative as simply ‘a told story’ owing to an etymological conjecture from the Latin *nārrāre* might be too broad) and it is not too restrictive. Moreover, it seems that Charon indicates clearly that she takes a narrative to be *defined* as a story with a teller, listener, time course, plot, and point. The characteristics of narratives she provides might be those characteristics which arise from stories meeting these other criteria, but I will use the definition that Charon provides.

Charon later seems to affirm this definition when she describes narrative knowledge: “If narratives are stories that have a teller, a listener, a time course, a plot, and a point, then narrative knowledge is what we naturally use to make sense of them” [5, p. 9]. She continues:

Unlike scientific knowledge or epidemiological knowledge, which tries to discover things about the natural world that are universally true or at least appear true to any observer, narrative knowledge enables one individual to understand particular events befalling another individual not as an instance of something that is universally true but as a singular and meaningful situation. Nonnarrative knowledge attempts to illuminate the universal by transcending the particular; narrative knowledge, by looking closely at individual human beings grappling with the conditions of life, attempts to illuminate the universals of the human condition by revealing the particular [5, p. 9].

There is much to unpack from these two sentences of Charon’s. If one ignores the talk of universal truth, Charon’s point in the first sentence is resonant: at least some types of knowledge are concerned with or aimed at truth. By contrast, what Charon calls narrative knowledge is aimed at elucidating the meaning of a narrative (perhaps to an individual). I find Charon’s references to universals and singularity somewhat mysterious and possibly confused. Plausibly, narratives can be about or experienced by groups of people, rather than an individual. Further, narratives might represent multiple, rather than single, situations. And moreover, the distinction between ‘nonnarrative’ and narrative knowledge seems a little steep—a charitable reading of scientific efforts probably interprets at least some scientific activity less so as ‘transcending the particular,’ rather more like characterizing causal relationships—studying causes and effects—and making tentative predictions about similar effects from similar causes. Charon’s claim that narrative knowledge ‘attempts to illuminate the universals of the human condition by revealing the particular’ thus seems in tension with the preceding sentence. Narrative knowledge, according to Charon, is not about universals (not universal truths anyway), yet it also attempts to illuminate universals of the human condition. To escape charge of contradiction, these ‘universals’ cannot be truths, leaving the question of what exactly they are. Additionally, ‘universals of the human condition’ might be empirically dubious (i.e., why should one think that they exist?).

Still, I think that there is something accurate inside Charon’s sentences, and one can access it by taking a modest approach that holds back on making broad generalizations (e.g., nonnarrative and narrative knowledges, universals and particulars). Thus, I will attempt to clarify and nuance Charon’s account to provide a more plausible view of narrative knowledge.

There are different kinds of knowledge. There is ‘knowledge-that,’ often called ‘propositional knowledge’ (e.g., knowing that a bike has two wheels). There is ‘knowledge-how,’ often called ‘technical knowledge’ (e.g., knowing how to ride a bike). I also think that there is a third kind of knowledge, which I call ‘knowledge-what-it’s-like,’ ‘phenomenal knowledge,’ or ‘experiential knowledge’ (e.g., knowing what it is like to be on a bike; see [9–12]) Charon seems to think that scientific and epidemiological knowledge fall under the ‘knowledge-that’ category—as these types of knowledge aim at truth values for propositions. Scientific and epidemiological knowledge are probably thus named because they come from scientific and epidemiological *studies*, respectively. So, it stands to reason that narrative knowledge is just that knowledge which comes from participating in the telling or receiving of a narrative. The more difficult question, then, is which of the three kinds of knowledge is narrative knowledge. It seems to me that Charon has two *prima facie* viable options: either narrative knowledge is ‘knowledge-that,’ through which a physician learns *that* a patient has experienced something, or narrative knowledge is ‘knowledge-what-it’s-like,’ through which a physician learns *what it’s like* to experience something a patient has experienced. I think that neither is satisfactory for Charon’s aims, which begins to unveil the deeper limitations of narrative medicine.

Suppose that narrative knowledge is meant to convey ‘knowledge-that.’ This proposal is the more modest of the two, given that it puts narrative knowledge in the position of conveying facts or information *about* the experiences of patients, rather than somehow conveying those experiences themselves. The examples Charon provides often suggest that ‘knowledge-that’ is what narratives provide. The first patient example she provides in the book is of a patient that Charon calls Luz, who provided a disability form that Charon begrudgingly signed. Charon later learned that Luz had been experiencing a range of difficulties, from financial pressure to sexual abuse, requiring disability income for her survival. This example is one that Charon presents as illustrating the merits of ‘narrative competence’ for physicians. Charon writes that learning Luz’s narrative made her more invested in the patient, enabling her to treat Luz with empathy. There is good reason to think that the narrative knowledge conveyed by Luz’s story is ‘knowledge-that.’ What Charon learned, after all, is *that* Luz experienced sexual abuse and *that* Luz had immense financial difficulties. She may have also learned *that* there was a kind of urgency to Luz’s request that she did not appreciate before, *that* Luz has been affected by her powerful experiences in an intimate way, or *that* Luz has feelings about her difficulties that would move Charon to action.

But if narrative knowledge is meant to convey propositional knowledge, it is no longer clear why narratives, the ‘stories with a teller, a listener, a time course, a plot, and a point’ are strictly necessary for the empathetic care of patients. After all, it seems that an empathetic interpreter could encounter several propositions about Luz (in no particular order and with no particular connection, e.g., “Luz’s sisters are experiencing sexual abuse,” “Luz is financially supporting five sisters,” “Luz is dependent on an abusive father and uncle for shelter and sustenance”) and treat Luz with empathy. Perhaps narratives provide some kind of glue to connect propositions and present them in a digestible form, but presumably physicians can be (or maybe have an *obligation* to) act empathetically when provided with information about a patient, even if those propositions are not organized into a narrative. Moreover, the difference between physicians who do and do not respond to these propositions with empathy might not simply be that the former approach these propositions from a narrative lens while the latter do not (as Charon seems to assume). Indeed, there are plausible structural forces (e.g., patient loads, insurance battles, administrative responsibilities, documentation burden, etc.) that actually lead to the deficits in empathetic care (which will be revisited in Sect. "Narrative-listening or active-listening?"). There are also other plausible internal factors that may be at work—consider Charon herself, who resolves to listen carefully to the narratives of patients to treat them with empathy. Perhaps the additional attention to the propositions in question, rather than the narrative structure that binds them, is what engenders this empathy. At any rate, a view in which narrative knowledge is ‘knowledge-that’ seems to betray Charon’s purposes—if propositional knowledge is what is conveyed by narratives, the version of narrative medicine one receives is deflated and readily replaced by whichever means, narrative or non-narrative, that physicians (at least those physicians resolved and otherwise lacking obstacles to be empathetic) use to uncover relevant facts from patients and/or their representatives (e.g., family, close friends), regardless of any underlying structure of these facts. Preferably, these means would involve empathetic, open, and active listening without preconception (though this aspect will be explored in Sect. "Narrative-listening or active-listening?").

Thus suppose, conversely, that narrative knowledge is meant to convey ‘knowledge-what-it’s-like.’ There is reason to believe Charon thinks that this experiential, rather than propositional, knowledge is what narratives convey, including her quotation of R.W.B. Lewis in saying: “Narrative deals with experiences, not in propositions” [13, p. 3]. Embracing this interpretation escapes the problems just mentioned with treating the knowledge that narratives provide as propositional knowledge.

However, other difficulties are introduced with this alternative proposal. It is unclear how hearing a narrative can convey experiential knowledge (i.e., ‘knowledge-what-it’s-like’), at least in any serious sense. Charon did not learn *what it is like* to be sexually abused or *what it is like* to have financial difficulties solely from engaging with Luz’s story. More importantly, she certainly did not learn *what it was like* for *Luz* (as Luz) to be sexually abused or have those financial difficulties (much as one cannot know what it is like to be a bat *as a bat*, but merely as oneself, see [9]). In other words, Charon cannot plausibly develop *experiential knowledge* of Luz’s experiences merely by hearing about them from Luz.

Other examples illustrate the difficulty as well. If I tell you the story of my having chemotherapy, you do not end up knowing what it is like to have chemotherapy, and certainly not knowing what it was like for me (*as me*) to have chemotherapy—you do not gain experiential knowledge of chemotherapy from hearing about my experience with it. You might have ‘knowledge-that’ *about* my ‘knowledge-what-it’s-like’ (e.g., you might end up knowing *that* chemotherapy was unpleasant for me or *that* chemotherapy caused me abdominal pain), but you cannot acquire knowledge of what it is like to undergo chemotherapy nor the experiential knowledge of the various pains involved in undergoing therapy from hearing my story.

The gulf between experience and storytelling cannot be stressed enough. There is no substantive sense through which someone can know what it is like to experience X by hearing about someone else’s narrative about experiencing X. Insisting that I know what it is like to be pregnant because a friend of mine told me about her pregnancy, no matter how detailed her telling is, would be absurd. A similar argument holds for ‘knowledge-how:’ Telling me about your riding a bike does not lead to my knowing how to ride a bike (even if it may facilitate my later learning to learn a bike in some way or another).

When one takes the microscope to narrative knowledge, it becomes difficult to see how it could function properly in Charon’s proposal. Either narratives are meant to convey ‘knowledge-that,’ making narratives a mere structural addition onto certain groups of propositions, or they are meant to convey ‘knowledge-what-it’s-like,’ which is impossible given that only experiences convey experiential knowledge. Thus, it is not clear that narrative knowledge is distinct from other kinds of knowledge, at least not in what the knowledge conveys.

However, perhaps one ought to take a step back. Perhaps this emphasis on narrative knowledge may appear tangential. Maybe narratives function in a different way, rather than providing some special kind of knowledge distinct from other kinds of knowledge. Here is one plausible proposal: Perhaps narratives structure propositions (i.e., ‘knowledge-that’) in a certain way that makes the content of these narratives more accessible, tapping into a distinctively human mode of knowledge transmission that engenders empathy within the patient-physician relationship. This proposal is considered next.

## Whose narrative?

In the previous section, I argued that Charon’s proposal of narrative knowledge seems confused—either narrative knowledge is supposed to be ‘knowledge-that,’ meaning that it does not amount to much over and above a particular structuring of propositions, or narrative knowledge is supposed to be ‘knowledge-what-it’s-like,’ at which point it becomes difficult to see how narrative knowledge could genuinely be conveying the experiential aspects of illness, at least in any substantive sense.

Perhaps instead one can try to defend Charon’s picture of narrative medicine by providing an alternative reading. What is crucial about narratives, so this reading holds, is not so much that a novel kind of knowledge is being conveyed, but rather that the knowledge is being conveyed in a particular *way*. Namely, narrative structure enables a deeper level of identification and empathy with patient experiences as opposed to a mere group of propositions that do not feature a teller, a listener, a plot, a time course, and a point.

Other commentators have found aspects of this proposal untenable, particularly in resisting that narratives have any essential, rather than merely contingent, centrality to illness experiences (or at least that this centrality is overstated [3, 4]). I also think that this proposal is ultimately dissatisfying, though I propose in this section that the way in which it is dissatisfying is instructive as to the core limitations of narrative medicine for serving patients in the way it aspires to.

Storytelling is, presumably, a very old mode of transmission between human beings. Sharing narratives may well be prehistoric—and thus, one might think that the narrative is the ‘secret sauce’ that makes for effective transmission of not only a series of propositions, but also for identification *with* the experiences of another. That is, by hearing a group of propositions structured into a narrative (e.g., “I was an avid runner in college, but then I broke my knee just before graduating. I delayed care but realized that I am feeling depressed without being able to run, so I am seeking care for my knee” versus “I am a runner,” “I broke my knee,” “I am depressed,” and “I am seeking care for my knee” as bare propositions presented to the physician), one is called to action in a stronger way. Charon indeed thinks physicians should be “moved by what they behold so that they can act on their patients’ behalf” [5, p. 3].

But I think that even this proposal is suspicious. Why should one think that physicians need narrative structures to properly empathize with patients (rather than, say, removal of administrative burdens or addition of more time with patients)? Plausibly, one would want physicians who would be empathetic toward speakers conveying propositions like, “I am worried I have cancer” that do not have a plot, a time course, or (perhaps) a point. One may even want physicians to be “moved by what they behold” so that they act, even if what they behold fails to be a narrative. I am inclined to think that if there is anything special about empathetic caretaking between a physician and a patient, it consists not in the narrative constructed, but in the direct attention and listening to the patient, no matter what form their report of illness takes.

Furthermore, if a narrative is needed to promote empathy, who is on the hook for constructing it? Is it the patient, who may or may not approach the world through the physician’s conception of narrative (perhaps entrenched in Western norms of literature and storytelling)? This option seems undesirable [2]: why burden the patient with telling a tale of their illness in order to receive empathetic care, especially if the patient is not particularly disposed to do so? A physician should be empathetic and willing to help a patient who says, “I think I broke my knee” just as urgently as a patient who tells a story about their long-standing passion for running. While a physician may be able to provide more emotional counseling to the latter patient, it should not be the responsibility of the vulnerable patient to effectively communicate the narrative to the physician or whether the physician receives the narrative; the physician is not owed the narrative by virtue of being a physician. In other words, the onus should not be on the patient to provide a narrative to receive empathetic care [2]. Rather, the physician should be able to employ other skills aimed at empathetic connection.

Another example may help illustrate the point. Suppose two patients come to your clinic with identical types of headaches. The first tells you that she struggled with substance use in high school and parts of college, before realizing that her love of studying philosophy was the missing ingredient to bringing fullness into her life. Philosophizing became a route for her emotional completeness, reducing her substance use to nothing. Her headaches, however, have begun to interfere with her thinking, making her philosophizing rather difficult. She worries that her substance use, which took years to overcome, might return because of her debilitating headaches. The second patient provides you with the following propositions (in a random order) with nothing further: “I have headaches,” “The headaches get in the way of my activities,” and “I’m worried that the headaches will lead to substance use.” The patient is not willing to tell you any other personal information about herself, either from tiredness, pain, or something else (that is not mistrust).^1^

I think that one should be equally compelled to empathize with both patients. Perhaps the narrative facilitates easier empathizing with the first patient, but the absence of a narrative (e.g., there is no time course or plot) in the second patient *should* not interfere with one’s drive to empathize with the patient. Perhaps one needs to employ certain unusual empathetic faculties to achieve this level of empathy, but the second patient’s treatment should not be less careful or thoughtful because they were unwilling to provide a narrative. After all, if a physician were criticized for treating the first patient with more empathy than the second, an excuse like “Well, the second one didn’t provide me with a narrative” would be coarsely unacceptable.

But if this is true, and physicians should be willing and able to provide empathetic care whether or not the patient is willing to construct a narrative for the physician, what is the point of the narrative in the first place? Perhaps it is a useful tool for exercising empathy when it is available, but it is far from essential in ensuring that the physician takes care of the patient. Imagine that the second patient arrives in such pain that she is unwilling to speak: certainly, no narrative is to be gleaned from this patient, but should she be treated with less empathy? Surely not. So, while a narrative might facilitate empathy between a physician and patient, it cannot be said to be *necessary* for empathy between a physician and a patient. Again, the onus should not be on the patient to construct and deliver a narrative of their experiences to receive empathetic care: the onus should be on the physician to care for the patient, however they may present.

But supporters of narrative medicine may respond that narrative medicine is no worse off for what I have argued above. They might suggest that it is the physician who develops the narrative, through reconstructive faculties of whichever components the patient is able to provide and careful imaginative faculties to ‘fill in the gaps.’ This would be the primary way in which a physician could end up with a narrative when a patient is unable to provide one. However, if the patient is not the constructor of their own narrative, a more unsettling alternative is presented. Imagine a patient in the psychiatric unit of a hospital who conveys the propositions: “I am being followed by the CIA,” “I was abused as a child,” and “I am the king of an alien faction.” If a psychiatrist were the one responsible for constructing the narrative from these propositions, one may have reasonable worries as to whether the physician would properly or accurately construct a narrative. For one, it is possible that some patients may not experience each aspect of their lives in a narrative structure, so imposition of a narrative structure alienates the patient’s experience from themselves [3]. Even if all patients *do* experience their illnesses as narratives, it is not clear (or likely) that the physician could entirely assume the patient’s lived experience to construct an accurate narrative. One should not and cannot expect that physicians know what it is like to experience their patient’s illnesses in order to construct an accurate narrative. And, even if they could, it is not clear what this narrative would provide over and above the relevant information communicated by the patient. That is, the most valuable information *about* the patient is likely the information *from* the patient. Whether it happens to be a narrative (a structured set of propositions), a set of propositions without a narrative, or anything else, the physician should be focused on *listening* to the patient, rather than *constructing* or *picking out* a narrative.

Of course, Charon (and other commentators) variously emphasize the role of listening in the patient encounter [5, 14–16]. However, this kind of listening is deeply entrenched within the exaltation of the narrative (i.e., listening is a means to construct, or more charitably, access, the narrative). Even Charon’s “radical listening” and “attentive listening” appear to be neatly tied to the objectified unit of patient stories [17, 18]. Charon, in a later work, favorably describes the listening she has in mind as “not unlike of close reading… the same attention to all features of the narrating are awakened” [19, p. 167]. She goes on to advocate for teaching close reading skills as an avenue to developing attentive listening skills [20].

But there is a critical difference (if not many) between books and patients: one is usually entitled to a story from the former, but we are not entitled to a story from the latter. However, if one insists on extracting a story from a patient who does not experience their own lives as a narrative commensurable with what the clinician takes to be a narrative (or lacks the energy or desire to construct one), one (once again) neglects the patient’s experience of their own illness.

The danger here is palpable: just as a clinician may hastily listen for whichever details confirm their diagnosis, a narrative medicine practitioner is at risk of listening for (or at least attributing higher significance to) whichever details conform to the narrative that they are trying to construct. Given that this emphasis on narrativity risks overriding the lived experiences of patients, it is clear that another method is needed.

This method, which I take to be the keystone of empathetic, patient-centered medicine, is the process of *actively listening* to the patient *with curiosity and without preconception*, rather than sifting for a narrative. It is up to the physician to reach within, empathize, and connect with the patient even if the patient does not present what the physician would count as a narrative.

## Narrative-listening or active-listening?

In the previous two sections, I discussed what I take to be deficiencies in the theoretical foundations of narrative medicine, namely in the failure of narrative knowledge to count as a substantive notion. I also claimed that narrativity as a requirement for empathetic connection with patients is dubious and that the elevation of the narrative to the object of pursuit in clinical encounters seems to miss the point of genuinely connecting with a patient, no matter how they present themselves.

In this section, I will consider improvements and alternatives to narrative medicine. That is, keeping in mind the limitations of narrative medicine noted thus far, I will attempt to detail ameliorative efforts for the enterprise of narrative medicine in a way that preserves patient-centered care. Should these efforts be unsatisfactory (as I think they are), I will entertain alternative frameworks from which to consider patient-physician relationships and encounters.

DasGupta (2008) has introduced “narrative humility” as an alternative to Charon’s “narrative competence” [21]. The basic idea is that physicians should be open to receiving narratives that are quite unlike what they may register or comprehend as a narrative. Rather, the patient’s narrative is something dynamic that a physician can humbly engage with, rather than apprehend as though an object.

I am sympathetic to this project, given that it takes a generous step in the right direction: narrative humility de-emphasizes the physician’s preconceived notions about narratives. Yet, following from the discussion in the previous two sections, I think that narrative humility does not go far enough. Baked into the structure of narrative medicine are unwarranted assumptions about the distinctiveness of narrative knowledge and a fundamental confusion about what narratives teach (i.e., ‘knowledge-that’ or ‘knowledge-what-it’s-like’) leading to disjunct possibilities, neither of which are satisfying. And, if narratives are simply tools to help physicians empathize with patients, it is unclear how narrative medicine could be inclusive of a wide range of patient experiences that cannot be neatly structured into narratives, or that at least require invasive restructuring of the patient’s words into a narrative that registers for the physician. So long as the narrative is elevated to the atom of clinical empathy, neither competence nor humility can truly be deployed toward inclusive, patient-centered care. If a patient presents with a telling of their illness without a plot, a time course, or a point, narrative humility will not rescue anyone; the narrative cannot carry the burden that narrative medicine hopes it can.

It might similarly be tempting to think that encouraging physicians to engage in reflective writing about patient encounters will improve patient care. After all, supporting physicians in thinking carefully about how they behave in clinical situations can lead to more empathetic care in future clinical encounters. Charon gives many such examples throughout her work [22, 23] and in her original book [5], in which she reflects formally upon a patient for whom she cared and conscientiously identifies weaknesses in her ability to connect with the patient, extracting actionable lessons from her clinical practice to benefit patients in future encounters.

I gather that these efforts are quite meaningful for physicians and may genuinely improve future patient care. Yet, it still seems to me that these strategies are physician-centered rather than patient-centered, though they have the capability to improve patient care as a welcome side effect of the process. If I emotionally harm a patient and reflect upon the experience later, I might learn to treat that patient better in the future, but the original harm is not undone. Moreover, if physicians continually engage in post hoc patchwork following missteps with patients, new patients remain at risk of (albeit reduced) harm primarily from whichever emotional oversight a given physician happens to have.

This claim is not to discredit the immense good that reflective writing can have for physicians. With increasing demands on physicians to treat more patients with less time and nearly insurmountable administrative burdens, a space for physicians to reflect on their encounters may be of immense importance for physician wellness. But once one accepts that one too hastily fetishized the narrative, one sees that the effort is exactly that: for physician wellness.

Another supplementary tool may be of use in advancing patient-centered care. This tool, I suggest, should be one that is fixated not on the narrative (which a patient may or may not provide), not centered around the physician’s experience of their patients, and ideally avoids burden on the patient to whatever extent possible while still providing patient-centered care.

The tool that fits the bill, I propose, is *active listening with curiosity and without preconception* (which I may refer to merely, hereafter, as ‘active listening’). Active listening is a general approach to communication that emphasizes effective and respectful avenues to mutual understanding [24]. Often, active listening is associated with conversational measures like asking clarificatory questions, reflecting and paraphrasing, maintaining focus on the interlocutor’s goals and concerns, etc., which are important components of clinical encounters.

However, these behaviors do not tell the full story about the potential for active listening to contribute to empathetic, patient-centered care. The point of active listening, beyond simply making the patient feel heard, is to *actually hear the patient*. That is, instead of sifting through the patient’s words for a narrative, instead of imposing a narrative structure of a patient’s description of their illness, and instead of elevating the patient’s story over the patient themselves, physicians should focus on accepting the patient, however they come, and being ready to treat them with empathy. This empathy should be extended to patients regardless of whether they present with a narrative, a collection of propositions, or even just silent behavioral cues or clinical signs.

Take Charon’s example of the patient Luz, who requests a signature on a disability form. Charon signs the form with some annoyance and later learns about the wide range of struggles that Luz had undergone, necessitating the disability payments. Suppose that Charon’s learning of Luz’s request instead prompted her to ask to what end Luz was seeking disability payments, which may have led to a later appointment with eventual disclosure, enhancing their relationship as patient and physician. Or suppose that Charon took a stance of humility and (given the brief nature of the encounter), provided reassuring words as she signed the form despite her dearth in understanding of Luz’s situation. Both alternatives could lead to Luz feeling acknowledged and respected by Charon.

Both alternatives are in the spirit of active listening. In the first, Charon requests more information so that she can better understand Luz’s situation. Note that the information Luz provides need not be in a narrative structure (e.g., it could be a series of propositions that convey her situation without a time course or a plot). In the second alternative, Charon has already done (some of) the active listening—she takes a stance of charity toward Luz, not presuming that Luz would make the request at such a time (i.e., without a scheduled appointment) unless there were serious urgency (which, after all, there was).

I turn to examples to demonstrate active listening because the concept is notoriously difficult to define in full. While active listening strategies are strongly suggested to be successful in enhancing conversational satisfaction and effectiveness in healthcare [25, 26], the set of exact techniques, beyond acknowledgement, nonverbal cues, and verbal responses like echoing, among others, is not universally concretized. However, the pleomorphism of active listening should be viewed as a feature, rather than a bug, of my view. Active listening is a responsive or reactive, rather than proactive, tool. Its scope should be as broad as the kinds of the actions or speech it is attending to, whether that be through denotative understanding of the words used or ‘reading between the lines.’ From physicians, active listening demands humility, careful reflection, humanistic commitment to charitable interpretation of the intentions of patients, and more. And unlike narrative medicine, active listening does not assume that the foundation of clinical empathy is the narrative. Active listening, in its truest form, is about removing unnecessary assumptions that bias the patient interaction, instead allowing the patient to present themselves in the way in which they will. The physician serves as an interpreter, and then optionally a confidant (though she is not entitled to this).

One of the goals of active listening, as I imagine is the case with narrative medicine, is to promote genuine, empathetic connection with patients. But what exactly is empathy, and how can one determine which tool might get one closer to empathy in clinical contexts?

## Empathy and active-listening

Unsurprisingly, there is a broad range of possible definitions of empathy. Some describe empathy as a neurologically hard-wired capacity in which the distress of another activates a resonant, if not reflective, response in the brain of the observer or listener [27]. While empathy shares an affective component with compassion, it has been sometimes described as motivated, in part, by duty or obligation, compared to compassion which is thought to motivated, in part, by love or altruism [28, 29].

Aside from the metaphysical understanding of empathy (e.g., its neurological correlates, its components, etc.) there is also the question of its role in healthcare. Some have advocated for so-called “detached concern,” in which concern is encouraged between clinicians and patients, albeit from a perspective of preventing a loss of objectivity or fatigue of emotional resources [30, 31]. Others have resisted this model, encouraging a combination of empathy and resilience (or robust replenishment of emotional resources) to better connect with and serve patients [32, 33].

Recent scholarship has argued for a preference toward empathy (or “empathetic concern,” which conveys both concern for patients while engaging the emotional resources of the clinician) in clinical settings, since it humanizes and strengthens the therapeutic relationship [34, 35]. Furthermore, narrative medicine has been proposed to enhance empathetic concern in physicians, and thus also patient’s perception of physician empathy, by enhancing focus onto the patient’s narrative [36].

I think that such a proposal is likely sound, though it is merely an instantiation of a more general phenomenon: When physicians engage in active listening, bolstered by a preconception-free and unassuming curiosity about the patient (that will usually inspire a particular line of questioning), the physician’s own empathetic faculties and the patient’s perception of these faculties being enacted are both activated. While in the case of narrative medicine, the line of questioning may be directed toward unearthing a narrative, the more general form of active listening that I propose, which does not require focus on a narrative but necessitates solely unbiased attention to the patient, encourages the same enactment of empathetic capacities. But rather than doing so by centralizing the narrative, I propose one does so by centralizing the patient. This amounts to a commitment to active listening without preconception and with curiosity, listening however the patient presents: willing and able to share a narrative, unwilling to share a narrative, or any other way, even including a patient’s silence or unconscious patients (i.e., listening for the quality of breathing, examining the body, etc.).

If active listening is the robust pathway to empathy that I claim it is, it is worth considering why efforts to prepare physicians for active listening might be unsuccessful. Some obstacles for active listening in clinical spaces might be attributed to gaps in medical training that emphasize techniques of active listening (e.g., eye contact, reiteration, careful listening before responding). However, I am inclined to think that a majority of the obstacles in the success of active listening have to do with the labor conditions of physicians.

Physicians have been estimated to spend one-quarter of their time on administrative duties [37]. The electronic health record is estimated to occupy 44.9% of physicians’ working hours [38]. One-third of the time spent with patients is spent simultaneously on the electronic health record [38]. Medical residents are trained in an environment where work weeks (including these administrative and documentation burdens) average or surpass 80 h [39]. Thus, woven into the very fabric of ‘physicianhood’ through medical training is a strand of urgency—not the urgency to connect earnestly with patients and listen actively to them, but to meet innumerable administrative and documentative tasks. Through the structure of medical training, patients are made secondary to the texts derived from them (e.g., patient notes, insurance billings, written narratives), making clear the emphasis of contemporary healthcare. While it is nearly impossible to obtain empirical data as to the effects of residency training structure, corporate healthcare systems, and administrative burden on empathetic care, one should be at least scrutinous of these features’ influence on humanistic medical care.

At the beginning of her book, Charon muses as to the source of the relative dearth of empathetic care among physicians compared to other health professions (e.g., nursing, pharmacy, psychological therapy). It seems plausible to me that the medical residency, with its intense workload (an 80-h work week in which only a roughly halved subset, a 40-h work week, is actually spent directly with patients) is one possible source. Charon correctly diagnoses these administrative and documentation burdens as siphons of empathy from the practice of medicine but arguably misses the point by prescribing narrative medicine as the antidote. Rectifying the labor conditions of the formative years of physicians so that active listening, humanistic empathy for patients, and deep humility are both implicitly and explicitly prioritized, is the cure.

The mechanism for rectifying these burdens in the face of a relatively small physician workforce with intensifying demands from complex insurance policies and corporate hospital structure (in which profit, rather than patient care, is a primary objective) is the purview of policymakers and healthcare leadership. Occupying neither of those roles, I regret that I am not in the position to provide solutions. However, I hope to have motivated a view by which active listening, and thus empathetic, patient-centered care, can be seen as plausible improvements to healthcare (given that the labor conditions of physicians in training and beyond allow for these strategies to take root).

## Conclusion

In this article, I looked closely at the assumptions behind narrative medicine, endeavoring to find the roles of narratives and narrative knowledge in the practice of medicine. I argued that narrative knowledge has, at best, an unclear standing as a distinct kind of knowledge (Sect. "What is a narrative? What is narrative knowledge?"). I claimed that neither of the two superficially plausible kinds of knowledge that narrative knowledge could be (i.e., ‘knowledge-that’ and ‘knowledge-what-it’s-like’) are satisfying. In one case, narrative knowledge becomes relatively deflated, conveying very little above propositions structured in a certain way. In the other case, narrative knowledge attempts to do too much, purporting to teach someone what it is like to experience potentially transformative illnesses and medical treatments without the listener having requisite experiences with those illnesses and treatments.

Still, in an attempt to rescue narrative medicine, I considered that narratives might be intended to enable humanistic connection with patient descriptions of illness experiences by structuring patient experiences in such a way that the physician can relate to them (Sect. "Whose narrative?"). However, I also dismissed this alternative as unsatisfying, since this imposition of narrative structure when it does not come naturally from the patient overrides the patient’s own description of their bodies and of their illnesses. That is, physicians should be moved to empathetic care whether or not a patient presents with a narrative at all, rather than a set of propositions or even non-verbal behavioral cues.

To address the problems that narrative medicine has, in my view, failed to, I proposed that active listening—with curiosity and without preconception—to patients, however they may present, is helpful for empathetic, patient-centered care (Sects. "Narrative-listening or active-listening?" and "Empathy and active-listening"). I noted that obstacles to achieving empathetic patient care through active listening include limited time with patients owing to administrative burdens for physicians, challenging labor conditions for physicians, and the general devaluing of patients (compared to patient data or texts from such data, including written narratives) engendered by a corporate, administratively-laden healthcare delivery system.

The discussion of narrative medicine is far from concluded. There are numerous further considerations in narrative medicine, including its ability or inability to handle patient-physician interactions of diverse racial, ethnic, religious, gender, etc., backgrounds. Further, a comprehensive program for active listening in clinical settings is a necessary, though not sufficient, step in implementing the ameliorative effort I sketched above. Moreover, and perhaps most troublingly, there is no straightforward solution to the larger structural problems that constrain the practice of medicine, progressively diminishing physicians’ capacity for holistic and humble engagement with patients. Still, I think that this avenue of adjustment, of improving labor conditions for physicians alongside larger reconsiderations of the appropriate goals for healthcare institutions, is a more plausible step in improving empathetic, patient-centered care than is narrative medicine. While narrative medicine may help physicians better understand themselves and incidentally improve care for patients in subsequent encounters, a deeper—directly to patients rather than indirectly from patients, patient-centered rather than merely physician-assistive, and preventative rather than post hoc—strategy is needed to truly hear those who entrust us with their care.

## Data Availability

There is no data associated with this manuscript.

## Footnotes

1 The burden should not be on the patient to justify their preference to not share certain personal information anyway. Presuming to have a right to every intimate detail of a patient’s life (or a narrative) simply in virtue of being a physician appears to me to be seriously misguided. Consider brief interactions, like emergency room visits, in which the length of time needed to build full rapport and trust is not always possible. Though the emergency physician is not entitled to every intimate detail of a patient’s life (e.g., a narrative), one still thinks that these physicians ought to treat that patient with empathy and care. The point of this example is to argue that the onus should not be on the patient to provide a narrative to receive empathetic care [2]. Rather, the physician should be able to employ other skills aimed at empathetic connection. These are discussed further in Sect. "Narrative-listening or active-listening?".
